# An attempt to improve the therapeutic effect of boron neutron capture therapy using commonly employed ^10^B-carriers based on analytical studies on the correlation among quiescent tumor cell characteristics, tumor heterogeneity and cancer stemness

**DOI:** 10.1093/jrr/rraa048

**Published:** 2020-06-30

**Authors:** Shin-ichiro Masunaga, Yu Sanada, Keizo Tano, Yoshinori Sakurai, Hiroki Tanaka, Takushi Takata, Minoru Suzuki, Koji Ono

**Affiliations:** 1 Particle Radiation Biology, Division of Radiation Life Science, Institute for Integrated Radiation and Nuclear Science, Kyoto University, Japan; 2 Particle Radiation Medical Physics, Particle Radiation Research Center, Institute for Integrated Radiation and Nuclear Science, Kyoto University, Japan; 3 Particle Radiation Oncology, Particle Radiation Research Center, Institute for Integrated Radiation and Nuclear Science, Kyoto University, Japan; 4 Kansai BNCT Medical Center, Osaka Medical College, Japan

**Keywords:** quiescent tumor cell, ^10^B microdistribution, tumor heterogeneity, cancer stemness

## Abstract

Based on our previously published reports concerning the response of quiescent (Q) tumor cell populations to boron neutron capture therapy (BNCT), the heterogeneous microdistribution of ^10^B in tumors, which is influenced by the tumor microenvironment and the characteristics of the ^10^B delivery carriers, has been shown to limit the therapeutic effect of BNCT on local tumors. It was also clarified that the characteristics of ^10^B-carriers for BNCT and the type of combined treatment in BNCT can also affect the potential for distant lung metastases from treated local tumors. We reviewed the findings concerning the response of Q tumor cell populations to BNCT, mainly focusing on reports we have published so far, and we identified the mode of BNCT that currently offers the best therapeutic gain from the viewpoint of both controlling local tumor and suppressing the potential for distant lung metastasis. In addition, based on the finding that oxygenated Q tumor cells showed a large capacity to recover from DNA damage after cancer therapy, the interrelationship among the characteristics in Q tumor cell populations, tumor heterogeneity and cancer stemness was also discussed.

## INTRODUCTION

The heterogeneity of the tumor microenvironment, including factors such as the oxygenation status, blood flow status and nutritional status in the tumor, creates a region of hypoxia, low blood flow and low nutrition. This can cause the tumor cells to enter a quiescent (Q) state, leading to low radio-sensitivity and reduced drug distribution, making the tumor resistant to cancer treatment. Further, the heterogeneity of the Q tumor cell population itself is also thought to be high, thereby making the therapeutic effect heterogeneous and increasing the possibility of recurrence after treatment [1, 2].

Boron neutron capture therapy (BNCT) has been employed in clinical research for >25 years at our institute. In BNCT, a neutron beam is delivered to the lesion tumor area following administration of a ^10^B-carrier agent, meaning that the outcome of BNCT will be influenced at the times of both ^10^B distribution and neutron beam irradiation [3, 4].

In order to enhance the therapeutic effect of BNCT, we examined various combinations of ^10^B-carrier administration and beam irradiation, using tumor-bearing mice whenever possible. Using a unified method for analyzing therapeutic effect, that can evaluate only the biological effect of α-rays and recoil lithium generated from the capture reaction between ^10^B and thermal neutrons [^10^B (n, α) ^7^Li], and can be also applicable in a treatment trial with newly synthesized ^10^B-carriers, treatment outcomes were rigorously evaluated. Based on the results, we made several attempts to determine the optimal BNCT implementation method [5].

Based on the findings obtained so far using our previously-proposed assay method that can simultaneously evaluate both local tumor response as a whole, including Q tumor cell response, and distant lung metastatic potential, the usefulness of several therapeutic combination methods for controlling local lesion tumors has been proposed and examined, aiming at optimizing cancer treatments including BNCT from the viewpoint of both controlling local tumor and suppressing the potential for distant lung metastasis. These studies afforded insights into the most effective combinations when performing BNCT using commonly-employed ^10^B-carriers [6].

Furthermore, based on the findings obtained using a unique method that combines the method for detecting hypoxic regions with the method we previously developed to enable selective detection of the response of Q tumor cell populations to cancer treatment, it was revealed that oxygenated Q tumor cells have a large capacity to recover from DNA damage after cancer therapy [7]. This characteristic of oxygenated Q tumor cells strongly supports a link to cancer stem cells, which are considered to be highly resistant to DNA damage and in a Q state. Therefore, the interrelationship between heterogeneity itself in Q tumor cell populations and cancer stemness has also been analyzed.

Mice used in all experiments were handled according to the Recommendations for Handling of Laboratory Animals for Biomedical Research, compiled by the Committee on Safety Handling Regulations for Laboratory Animal Experiments at Kyoto University.

## Establishment of a method for detecting the response of Q tumor cell populations in solid tumors to DNA-damaging treatment

Human solid tumors are thought to contain moderately large fractions of Q tumor cells that are out of the cell cycle and have ceased cell division, but are viable compared with the established experimental animal tumor lines employed in various oncology studies. The presence of Q cells is probably due, in part, to hypoxia and the depletion of nutrition in the tumor core, which is another consequence of poor vascular supply. As a result, Q cells are viable and clonogenic, but have ceased cell division. In general, radiation and many DNA-damaging chemotherapeutic agents kill proliferating (P) tumor cells more efficiently than Q tumor cells, resulting in many clonogenic Q cells remaining following radiotherapy or chemotherapy [8]. It is therefore harder to control Q tumor cells than to control P tumor cells, and many post-radiotherapy recurrent tumors are thought to result partly from the regrowth of Q tumor cell populations that could not be sufficiently killed by radiotherapy [9]. Further, sufficient doses of drugs cannot be distributed within Q tumor cell populations mainly due to heterogeneous and poor vascular distribution within solid tumors. Thus, one of the major causes of post-chemotherapy recurrent tumors was also thought to be an insufficient dose distribution in the Q cell tumor fractions [10, 11]. Our method for selectively detecting the response of intratumor Q cells has made it possible to evaluate the usefulness of various modalities for cancer therapy in terms of effectiveness on the intratumor Q cell population [12]. Based on the characteristics of the intratumor Q-cell responses to various DNA-damaging treatments, more effective and useful modalities for cancer therapy can be developed.

Q cells are operationally defined as those cells not in active proliferation during measurement [1, 13]. We consider the term ‘quiescent’ to include all tumor cells that are out of the cycle, irrespective of the reason. The Go state, in contrast, is confined to viable cells that are out of the cycle under normal physiological conditions, and that can be recruited into active proliferation by an appropriate stimulus [14]. The best examples of these cells are found in normal intact tissues (liver, salivary gland, etc.). However, the response of intratumor Q cells *in vivo* had not been directly detected until we developed the method described below.

**Fig. 1.**
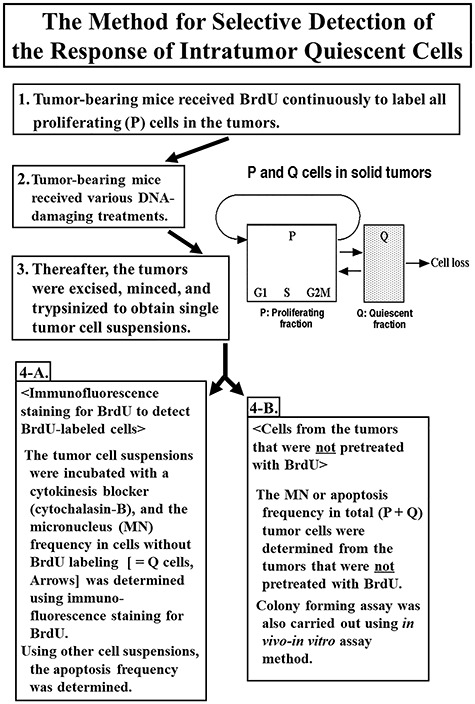
Flow diagram summarizing the procedures for determining the responses of quiescent and the total tumor cells, and the concept of proliferating and quiescent cells in solid tumors.

Tumor-bearing mice were treated with various DNA-damaging treatments after continuous labeling with 5-bromo-2′-deoxyuridine (BrdU) for >5 days to label all P tumor cells in solid tumors. The tumors were then excised and trypsinized. The obtained tumor cell suspensions were incubated with cytochalasin-B (which blocks cytokinesis) for 48–72 h, and the micronucleus (MN) frequency in these cells without BrdU labeling was determined using immunofluorescence staining for BrdU. This MN frequency was then used to determine the surviving fraction (SF) of the BrdU-unlabeled cells from the regression line obtained between the MN frequency and the SF determined for the total (= P + Q) cells in the tumor. A cell-survival curve was thereby determined for cells not labeled by BrdU, which could be regarded for all practical purposes as the Q cells in a solid tumor (Fig. 1) [12, 15].

When cell division is disrupted, or the chromosomes are broken or damaged by chemicals or radiation, the distribution of genetic material between the two daughter nuclei during cell division is affected and pieces or entire chromosomes fail to be included in either of the two daughter nuclei. The genetic material that is not incorporated into a new nucleus forms its ‘micronucleus’. Thus, the frequency of MN formation reflects the genotoxicity of the chemical compound or radiation very well. BrdU-labeled tumor cells were detected by indirect immunofluorescence staining using a monoclonal anti-BrdU antibody and a fluorescein isothiocyanate (FITC)-conjugated antimouse IgG antibody. To distinguish the tumor cells stained with green-emitting FITC and observe them separately, cells on the slides were treated with red-emitting propidium iodide as background staining and monitored under a fluorescence microscope. The MN frequency in cells not labeled with BrdU could be examined by counting the micronuclei in the binuclear cells that showed only red fluorescence. The MN frequency was defined as the ratio of the number of micronuclei in the binuclear cells to the total number of binuclear cells observed [15].

Meanwhile, analytical detection based on the apoptosis frequency was carried out as follows. At the optimal time after DNA-damaging treatment, solid tumors were excised, minced and trypsinized. The obtained tumor cell suspensions were fixed and the apoptosis frequency in cells without BrdU labeling was morphologically determined using immunofluorescence staining for BrdU. The optimal time for tumor excision after each DNA-damaging treatment was determined in advance such that the maximum value of apoptosis frequency could be histologically observed. Thereafter, it was clarified that the apoptosis frequency, as well as the MN frequency, can be applied to our method for measuring the Q cell response to DNA-damaging treatment, such as radiation, in solid tumors. Similar radiobiological findings for intratumor total and Q cells were obtained in our research, whether based on the MN frequency or the apoptosis frequency [16].

Incidentally, it was found that Q tumor cells have lower clonogenicity than P tumor cells [17], and that some of the Q tumor cells can shift to a cell fraction that leads to a situation called ‘cell loss’, the extent of which depends on the type of tumor [13, 18]. However, depending on the type of DNA-damaging treatment to tumors, there are certainly tumor cells that shift from Q to the P status through the treatment [19]. Therefore, it is still important to control treatment-resistant Q tumor cells. Furthermore, in carrying out this assay, essential BrdU administration did not cause significant adverse events such as growth inhibition in the growth of transplanted tumors.

## Directly-demonstrated characteristics of Q tumor cell populations

### Directly-detected response of Q tumor cell populations to conventional cancer therapy

As a result of the analysis using the method to selectively detect the response of Q tumor cell populations, the following findings were obtained: Q tumor cell populations in the local solid tumors are more resistant to radiation than the total tumor cell population as a whole and have a greater capacity for recovery from DNA-damage and a larger hypoxic cell fraction (HF) than the total tumor population [12]. The hypoxic region of a Q tumor cell population is mainly made up of chronic hypoxia caused by being far from tumor blood vessels, as opposed to acute hypoxia caused by uncontrolled and spasm-causing tumor blood vessels [1, 8, 9, 20]. Further, each of the above characteristics is observed regardless of the p53 status of the tumor cells, and rather more clearly in the wild-type than in the mutant-type tumors [21]. In addition, the colony forming ability of Q tumor cells is smaller than that of P tumor cells [17], and recruitment of Q to P status in tumor occurs after low energy transfer (LET) irradiation [19].

In response to the administration of chemotherapeutic agents, the chemo-sensitivity of Q tumor cells to cisplatin was lower than that of the total tumor cell population. However, when cisplatin was used, the sensitivity difference between total and Q cells was greater than that for X-ray irradiation [22]. This difference is probably attributable to the uneven distribution of cisplatin in the Q cell population due to the heterogeneity of the tumor vasculature, compared with the homogeneous delivery of X-rays throughout solid tumors [22, 23].

Meanwhile, the sensitivity of Q tumor cells to tirapazamine (TPZ), a well-known bioreductive agent that preferentially kills hypoxic cells, was greater than that of the total tumor cells. Following TPZ treatment, Q cells showed greater recovery capacity than the total cells. However, TPZ treatment caused a much smaller recovery capacity in both the total and Q cells [24]. This was compatible with Brown’s previous report showing that DNA breaks induced by TPZ are repaired much less readily than those produced by X-irradiation, because active TPZ radicals are produced at high local concentrations by activating enzymes close to the DNA [25]. In addition, two fractionated TPZ administrations and determination of the P cell ratios in the tumors at the time of the second administration indicated that the use of TPZ in the treatment of solid tumors causes a shift from the P to Q state i*n vivo* [26]. With regard to the oxygenation status in squamous cell carcinoma VII (SCC VII) solid tumors after drug administration, TPZ and cisplatin reduced the HF after treatment, especially in Q cells; this tendency was particularly marked with TPZ. In contrast, bleomycin increased the HF after treatment. Both reoxygenation following γ-ray irradiation or bleomycin treatment and a subsequent return to the pretreatment levels of HF following TPZ or cisplatin treatment (rehypoxiation) occurred more rapidly in acute hypoxia-rich total cells than in chronic hypoxia-rich Q cells [27].

Heat treatment has been demonstrated to be effective as an adjuvant modality to radiotherapy. Laboratory experiments showed that heating for 30–60 min at a relatively high temperature, i.e. >43–44°C, damages intratumor blood vessels and kills tumor cells. In addition, hyperthermia causes direct cellular radio-sensitization [1, 8, 28–30]. However, the currently-available heating devices have proved ineffective in raising the temperature of human tumors sufficiently to cause vascular damage, kill tumor cells and directly radio-sensitize the tumor cells. Previous clinical studies in which a heat treatment was shown to improve the effectiveness of radiotherapy suggested that hyperthermia might improve tumor oxygenation and thus indirectly radio-sensitize tumors through an increase in the tumor blood flow [29–31]. According to our reports, hyperthermia at mild temperatures (MTH) without direct cytotoxicity or direct radio-sensitization effects (40.0°C, 30–60 min) before irradiation decreased the HF in SCC VII tumors, even when combined with the administration of an acute hypoxia-releasing agent nicotinamide (NA). However, MTH did not decrease the HF when the tumor-bearing mice were placed in a circulating carbogen (95% O_2_/5% CO_2_) chamber, where chronic hypoxia is oxygenated. Therefore, MTH was thought to preferentially oxygenate the chronically-induced HF [32]. In addition, MTH decreased the HF of chronic hypoxia-rich Q cells of SCC VII tumors more markedly than the acute hypoxia-rich total tumor cell population, and the minimum values of HFs of both total- and Q-cell populations were obtained 6 hours after MTH. Two days after MTH, the HF of the total tumor cells returned almost to that of unheated tumors. In contrast, the HF of Q cells did not return to the level of unheated tumors until 1 week after MTH [33]. These findings also indicate that MTH could preferentially oxygenate chronic hypoxia-rich Q cell fractions in the tumors. Further, the time courses of changes in the decrease in the HFs of total and Q cell populations after MTH suggested that irradiation within 12 h after MTH might be a promising therapeutic method for controlling radio-resistant Q tumor cells, especially when it is difficult to elevate the tumor temperature enough to cause vascular damage, kill tumor cells and directly radio-sensitize the tumor cells within solid tumors. Actually, this treatment sequence for thermo-radiotherapy was proposed at several international and domestic scientific meetings, especially related to radiation oncology.

### Directly-detected response of Q tumor cell populations to neutron capture therapy

In our animal studies on intratumor Q-cell responses to a neutron capture reaction, ^10^B-boronophenylalanine (BPA) and ^10^B-sodium mercaptododecaborate (BSH), which are conventional ^10^B-carriers in clinical BNCT, were used for neutron capture therapy (NCT) [3, 4, 34]. Without the ^10^B-carriers, although Q cells showed lower sensitivity than the total cells, neutron irradiation reduced the difference in the sensitivity to γ-rays between the total and Q cells. With ^10^B-carriers, even when the ^10^B concentrations within solid tumors at the time of reactor neutron irradiation were similar to each other, BPA increased the sensitivity of the total cells to a greater extent than BSH. However, the sensitivity of Q cells treated with BPA was lower than that in BSH-treated Q cells. The difference in sensitivity between the total and Q cells was greater with ^10^B-carriers, especially with BPA [35, 36]. Q cells showed greater recovery capacity than the total cells after neutron irradiation as well as after γ-ray irradiation. γ -Ray irradiation and neutron irradiation with BPA induced larger recovery capacities in each cell population. In contrast, thermal neutron irradiation without the ^10^B-carrier induced the smallest recovery capacity in both cell populations. The use of a ^10^B-carrier, especially BPA, resulted in an increase in the recovery capacity in both cell populations, and made the recovery patterns of the two cell populations look like those induced by γ-ray irradiation [37]. In both the total and Q tumor cells, the HFs increased suddenly immediately after neutron irradiation. Reoxygenation after each neutron irradiation occurred more rapidly in the total cells than in the Q cells. In both cell populations, reoxygenation appeared to be rapidly induced in the following order: neutron irradiation without ^10^B-carriers > neutron irradiation following BSH injection > neutron irradiation following BPA administration > γ-ray irradiation [38]. Furthermore, γ-ray irradiation as the second irradiation following initial thermal neutron irradiation and continuous BrdU labeling until the time of the second irradiation, indicated that the use of the ^10^B-carrier, especially BPA, in thermal neutron irradiation causes recruitment from the Q to the P population [39]. These findings suggested that thermal neutron irradiation without a ^10^B-carrier reduces the difference in sensitivity between the total and Q cells, and that the ^10^B from BPA is more dependent on the drug-uptake potential of tumor cells than that from BSH, because P cells are thought to have a greater potential for drug uptake due to their aerobic and well-nourished condition, probably as a result of their rich and homogeneous vascular distribution [1, 2, 8–11, 15].

Concerning newly-developed ^10^B-carriers, therapeutic effects of α-amino alcohol para-boronophenylalaninol [40] and its enantiomers [41], 2-nitroimidazole-^10^B-carriers [42], hypoxia-oriented bioreductive agent-BSH hybrid compounds [43, 44], transferrin-pendant-type polyethyleneglycol liposomes encapsulating ^10^B-decahydrodecaborate (GB-10) or BSH [45], and a novel ^10^B-carrier conjugated with a cyclic RGD (arginine-glycine-aspartic acid) peptide [46] were analyzed and examined on both total and Q tumor cell populations. Incidentally, the cyclic RGD peptide specifically binds to αvβ3 integrin, which is known to be overexpressed on activated endothelial cells of growing vessels and also on melanoma, glioma, lung, ovarian and breast cancer cells [47]. The applicability of combination with the vascular targeting agent ZD6126 in BNCT was also evaluated using our method for selectively detecting the response of the Q tumor cell population [48]. However, adverse events of these newly developed ^10^B-carriers in normal tissues have not yet been analyzed in detail, a situation that must be clarified in the future.

With regard to gadolinium neutron capture therapy, water-soluble non-enriched gadolinium-containing contrast medium for magnetic resonance imaging (MRI) was not suitable as a neutron capture carrier in NCT, because non-enriched gadolinium in the form of a clinically used non-ionized contrast agent for MRI was too water-soluble to be retained in tumor tissue for a sufficient period during neutron irradiation [49]. Incidentally, gadolinium neutron capture reaction [^157^Gd(n, γ e^−^)^158^Gd] results in the emission of γ-rays with a maximum energy of 7.9 MeV, followed by a series of secondary γ-rays and 29–180 keV internal conversion electrons [50]. In such gadolinium neutron capture therapy, the internal conversion electrons and Auger electrons are thought to play important roles in microscopic energy deposition; these electrons are responsible for ~15% of the total absorbed dose in gadolinium-treated tumors [51].

### An attempt to enhance the sensitivity of Q tumor cell populations

Solid tumors, especially human tumors, are thought to contain a high proportion of Q cells. In the solid tumor core, hypoxia and the depletion of nutrition, partly due to poor vascular supply, are thought to cause the Q status of tumor cells. It has been reported that Q cells have lower sensitivity to irradiation and anticancer drugs than P cells in solid tumors *in vivo* [1, 2, 8, 9, 15]. This means that more Q cells can survive after anticancer treatment than P cells. Consequently, the control of Q cells has a marked impact on the outcome of anticancer therapy. Based on the above characteristics of Q cells within solid tumors, the following methods for enhancing the response of intratumor Q cells were proposed.

Based on the idea that control of the therapy-resistant Q tumor cell population consisting of a large hypoxic region must have a beneficial effect on controlling the local tumor as a whole, it was shown that a hypoxia-specific cytotoxin such as TPZ can be used to kill Q tumor cells regardless of the p53 status of tumor cells. TPZ has a strong cell-killing effect on the whole tumor, including the Q tumor cells [23, 52–54]. Meanwhile, since MTH improves blood flow in the tumor and relieves the chronic hypoxic areas that occur consistently depending on the distance from the blood vessel, it was also found that the use of TPZ combined with MTH further enhances the cytocidal effect on tumor cells, especially Q tumor cells, which are very resistant to conventional cancer therapy, again independently of the p53 status of the tumor cells [52–55]. Severe hypoxia is not required and the level of hypoxia found in many human tumors is sufficient for the toxicity of TPZ. It was shown that cells intermediate in oxygenation can influence tumor response to radiation and hypoxic cell cytotoxins, and such cells constitute a significant proportion of solid tumors. This suggested that MTH might change some chronically hypoxic fractions to a level intermediate between fully oxygenated and hypoxic through an increase in tumor blood flow, and at the same time distribute higher doses of TPZ and kill cells at these intermediate oxygen tension [23]. In contrast, the cell killing effect on acute hypoxic cells transiently occurring in various parts of the tumor due to sudden spasms caused by incomplete innervation of the tumor blood vessels could be achieved through continuous as opposed to single administration of TPZ [55, 56]. At present, it is thought that continuous TPZ administration combined with MTH, which can overcome even the decrease in radio-sensitivity due to low dose-rate irradiation, together with anticancer therapy, can most effectively control the local tumor as a whole, including the Q tumor population [57]. It can also be expected that the tumoricidal effect due to combination treatment with both continuous TPZ and MTH in low LET radiation therapy can be enhanced to a level comparable to particle radiation beam therapy [54, 56–60].

In current clinical BNCT, only BPA and/or BSH are used, mainly due to the toxicity of many newly-synthesized ^10^B-carriers [4]. It has been found that in the hypoxic tumor cells abundant in Q tumor cell populations, ^10^B from BPA is very hard to distribute and the amount of distribution itself is small, but that ^10^B from BSH is relatively more uniformly distributed in hypoxic areas within solid tumors. In addition, this characteristic related to ^10^B distribution from the ^10^B-carriers was more clearly shown in the tumors with wild-type p53 than mutant-type p53 status [61, 62]. Since p53 status is reported to be often mutated in malignant tumors, a relatively even distribution of ^10^B in tumors with mutant-type rather than wild-type normal tissue can be expected, leading to the usefulness of BNCT in the treatment of malignant tumors [63]. Actually, it was shown that in BNCT the combination of both continuous TPZ administration and MTH at the time of administration of these ^10^B-carriers can more effectively enhance the control of the local tumor as a whole, including the control of Q tumor cells, than any other combination treatment design [40, 41].

However, we have not paid special attention to the effect of these combined treatments on normal tissues, and the results of evaluating the effects are not available. Since there is no direct cell-killing effect by MTH level heating, MTH is considered to cause almost no adverse events. On the other hand, with regard to TPZ, the amount used is close to one-quarter to one-tenth of the LD50 (mean lethal dose) of the experimental mice employed, thus there is a possibility that the effect on normal tissues cannot be completely ignored.

## Evaluation of distant lung metastatic potential from treated local tumors

**Fig. 2.**
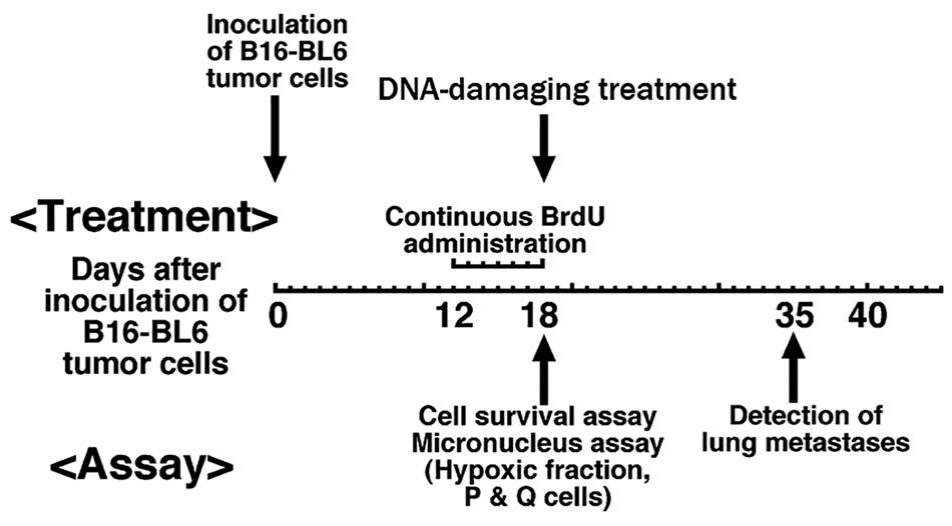
Flow diagram summarizing the experiment for determining the responses of quiescent and total tumor cells and the potential of distant lung metastases from irradiated local tumors.

Using an assay that can simultaneously evaluate both local tumor control and distant lung metastasis through combining our method for selectively detecting the response of Q tumor cells with that for detecting distant metastasis, the effect of manipulating the intratumoral oxygen tension for improving local tumor control on distant metastatic potential from the local tumor was examined. In particular, distant metastatic potential was detected by the method described below. Seventeen days after irradiation (i.e. 35 days after the inoculation of tumor cells), the tumor-bearing mice were sacrificed, and their lungs were removed, briefly washed with distilled water, cleaned of extraneous tissue, fixed in Bouin’s solution overnight, and stored in buffered 10% formalin until macroscopically visible metastases were counted under a dissection microscope (Fig. 2) [64, 65].

It was clarified that, under irradiation with conventional low LET radiation, administration of NA that releases acute hypoxia in the tumor [64, 65], continuous TPZ administration [66], or fractionated administration of bevacizumab [67], wortmannin, a non-specific covalent inhibitor of phosphoinositide 3-kinases [68], or thalidomide [69] can effectively suppress intratumoral heterogeneity represented by an acute hypoxic region showing dynamic spatiotemporal fluctuation, leading to reduction of lung metastatic potential from local tumor. In BNCT, BPA-BNCT rather than BSH-BNCT, especially when combined with NA [70] or fractionated administration of bevacizumab [71], could efficiently suppress distant metastasis from treated local tumor.

Controlling the intratumor Q cell population, which is resistant to conventional cancer therapy and has large chronic hypoxic regions, greatly affects the control of local tumors as a whole. Therefore, the oxygenation of chronic hypoxic regions, such as through MTH, is thought to be important for local tumor control. Meanwhile, with the aim of suppressing lung metastatic potential from treated local tumors, it is effective to oxygenate the acute hypoxic region using NA administration or continuous TPZ administration. This means that it is still important to control both acute and chronic hypoxic regions in solid tumors to improve the outcomes of cancer therapy.

In addition, in BNCT with two commonly-used ^10^B-carriers, BPA and BSH, the usefulness of the combination of continuous TPZ administration and MTH, which was considered to be the most effective combination design so far for enhancing local tumor control, including the control of Q tumor cells with conventional cancer treatment, was evaluated in terms of suppressing distant lung metastatic potential from treated local tumors, and compared with that of a combination design with NA administration that can also oxygenate acute hypoxia in tumors. The combination with continuous TPZ administration and MTH showed a significantly higher control rate of local tumors than that with NA, much higher than that with TPZ only or MTH only treatment [6]. Further, it was also found that distant lung metastatic potential was more effectively suppressed by the combination with continuous TPZ administration and MTH than with NA only, with TPZ only or with MTH only treatment. [6]. The combination treatment design with both continuous TPZ and MTH was shown to be useful and promising in BNCT, not only in controlling local tumors, but also in suppressing distant lung metastasis from treated local tumors. When performing BNCT using a conventional ^10^B-carrier, BPA or BSH, the combination of continuous TPZ administration and MTH is currently considered to be more effective and promising than any other combination design, from the viewpoint of both controlling the local tumor and suppressing distant metastatic potential from the treated local tumor.

Regarding the effect of these treatments, which are used in combination when performing DNA-damage treatment, on normal tissues, the doses of NA used and MTH employed are within the range that causes almost no adverse events. On the other hand, as mentioned above, TPZ was used in an amount close to one-quarter to one-tenth of the LD50 of the experimental mice, thus it is possible that the effect on normal tissues cannot be completely ignored.

## Characteristics of pimonidazole-unlabeled, probably oxygenated, Q tumor cell populations

**Fig. 3.**
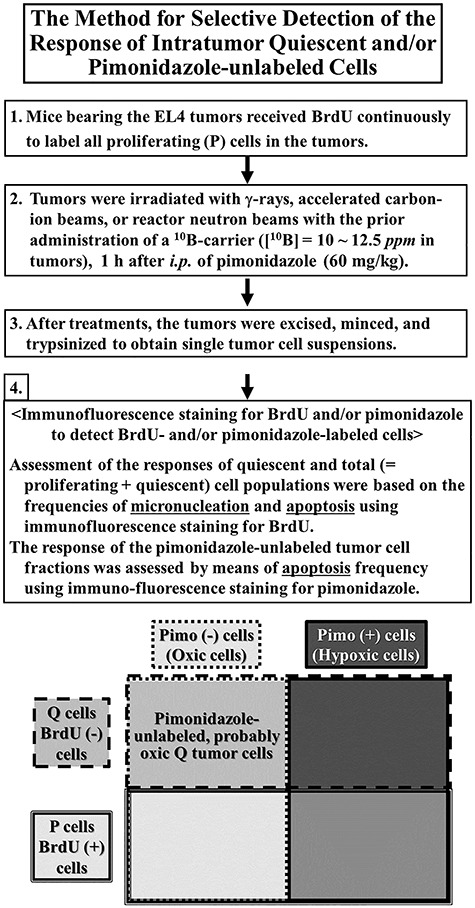
Flow diagram summarizing the procedures for determining the responses of pimonidazole-unlabeled oxic quiescent tumor cells, and the concept of pimonidazole-unlabeled oxic cells in solid tumors.

The characteristics of oxygenated Q tumor cell populations were analyzed using a unique method that combines a method for detecting hypoxia using pimonidazole and anti-pimonidazole antibody with our method for selectively detecting the response of Q tumor cells to cancer treatment. Pimonidazole is a 2-nitroimidazole with hypoxic selectivity and potential radio-sensitizing activities, and is used as a hypoxia marker for complementary studies of tumor hypoxia. After labeling all P cells in tumors by continuous administration of BrdU, tumors were irradiated 1 h after pimonidazole administration. Assessment of the responses of the Q and total cell populations were based on the frequencies of micronucleation and apoptosis using immunofluorescence staining for BrdU. The response of pimonidazole-unlabeled (i.e. probably oxygenated at irradiation) tumor cell fractions was assessed by means of apoptosis frequency using immunofluorescence staining for pimonidazole (Fig. 3) [7].

The Q tumor cell population showed greater capacity to recover from DNA damage than the total tumor population, while oxygenated Q tumor cells showed even greater recovery capacity. This characteristic of oxygenated Q tumor cells strongly supported an interrelationship with cancer stem cells, which are considered to be highly resistant to DNA-damaging treatment and in a Q state (Fig. 5) [7]. It is true that these findings were applicable to the solid tumors consisting of p53 wild-type tumor cells, but they were not always applicable to the solid tumors consisting of p53 mutated tumor cells because the recovery capacity from DNA damage was too small to detect in tumors consisting of p53 mutated tumor cells [72–75].

Meanwhile, this decrease in radio-sensitivity was suppressed following carbon ion beam and neutron beam-only irradiation. In boron neutron capture reactions, the use of a ^10^B-carrier, especially BPA, enhanced the sensitivity of the pimonidazole-unlabeled, probably oxygenated, cells more clearly in the Q cells than in the total cells [76]. We also found that continuous administration of TPZ [77] and fractionated administration of wortmannin [78], both of which release acute hypoxic regions, or metformin, which is one of the biguanide drugs used as an antidiabetic agent and widely used as the first-line medication for the treatment of type 2 diabetes, [79], efficiently suppressed this decrease in radio-sensitivity in oxygenated Q tumor cell fractions derived from recovery from DNA damage caused by γ-ray irradiation. Interestingly, this finding appeared to suggest that manipulation to mitigate heterogeneity within solid tumors, such as by release of acute hypoxia, may have the potential to suppress some of the characteristics of cancer stemness. Actually, a positive association between stemness and multiple metrics of intratumoral heterogeneity across cancers was recently reported [80]. Given recent work linking increased intratumoral heterogeneity with decreased immune cell infiltration, one could speculate that cancer stemness might contribute to intratumoral heterogeneity by both increasing the replicative capacities of individual tumor clones and shielding antigenic clones from elimination by the immune system. This means that in tumor cells, cancer stemness positively correlated with higher intratumoral heterogeneity, and that potential mechanisms of cancer stemness-associated immunosuppression were certainly observed and recognized [80, 30].

In recent years, immunotherapy has been widely praised, but its curative effect is still limited by tumor immune escape. Tumor immune escape refers to tumor cells that can avoid recognition and attack by the immune system by changing themselves or their microenvironment. The complex network of the tumor microenvironment significantly weakens the efficacy of immunotherapy, making it difficult to initiate immunotherapy in solid tumors. A crucial feature of the microenvironment that promotes tumor immune escape is the lack of tumor antigen recognition and antitumor T-cells [29, 30].

**Fig. 4.**
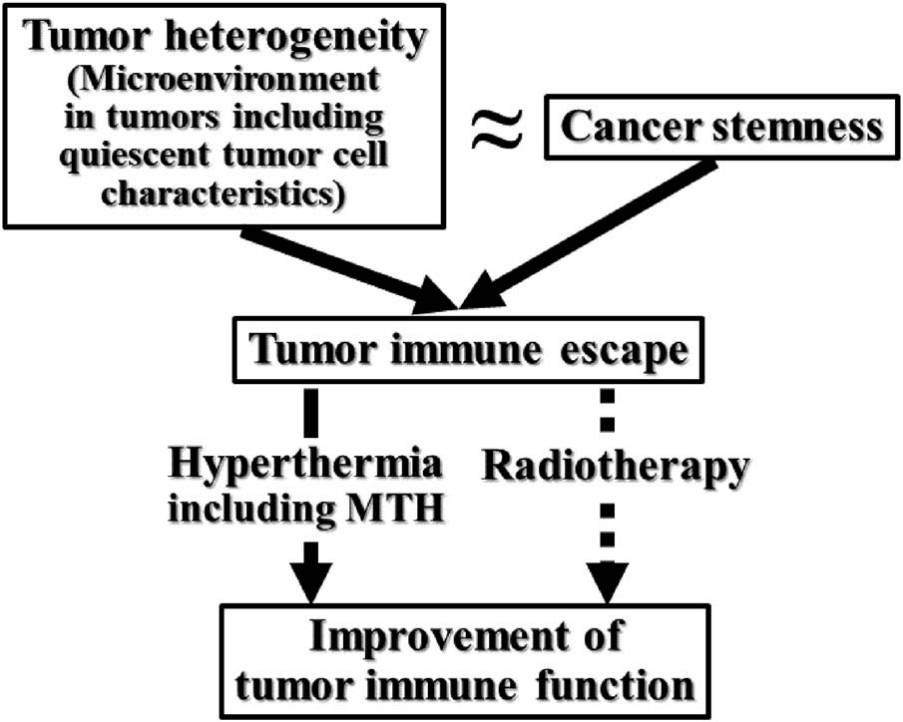
Diagram summarizing the correlation among tumor heterogeneity, cancer stemness and quiescent tumor cell characteristics taking into account the relationship with the effect of hyperthermia or radiotherapy on the immune system.

Hyperthermia can increase the trafficking of immune cells across the tumor vascular barrier to access the tumor and immune organs. This process probably occurs through elevated integrin- and selectin-mediated adhesion, stable binding to intercellular adhesion molecule-1, as well as increased endothelial cell activation and permeability. Through the application of hyperthermia, including MTH, tumor antigens are released by extracellular heat shock proteins during the process of tumor cell necrosis to activate antitumor immunity. This induced immunity has been further demonstrated to contribute to the control of the recurrence and metastasis of tumors [29]. On the other hand, although radiation was reported to augment microenvironment immunosuppressive effects through inducing apoptotic tumor cells and increasing the expression of transforming growth factor-β (TGF-β) or immune suppressive checkpoint molecules such as programmed death-ligand 1 (PD-L1), it is also accepted that radiotherapy also induces immune activation. After radiotherapy, the expression of major histocompatibility complexes, stress ligands, adhesion molecules, death receptors and ligands increase on tumor cells. Further, radiotherapy causes different cell-death modalities, such as apoptosis, necrosis, mitotic catastrophe or senescence. This leads to the spatiotemporal release of damage-associated molecular patterns that attract and activate cells of the innate and cells of the adaptive immune system [30] (Fig. 4).

In the future, we also would like to analyze more closely the correlation between cancer stemness and intratumoral heterogeneity taking into account the relationship with Q tumor cell characteristics.

## Future prospects

As a representative of heterogeneity in tumors, we analyzed the characteristics of the Q tumor cell population, including the oxygenated Q tumor cell population which has a large capacity to recover from DNA damage after cancer treatment and suggests an interrelationship with cancer stem cells. So far, we have analyzed the relationship among characteristics of Q tumor cell populations, cancer stemness and tumor heterogeneity. We hope that improved cancer treatment designs for localized lesion tumors based on the findings obtained from these analytical studies will contribute to further optimization of cancer therapy including BNCT, from the viewpoint of simultaneously controlling local tumors and suppressing distant lung metastatic potential.

## CONFLICT OF INTEREST

None declared.

## FUNDING

This study was supported in part by a Japan Society for the Promotion of Science (JSPS) KAKENHI Grant-in-aid for Young Scientists (04857111), Scientific Research on Priority Areas (2) (09255229), Scientific Research on Specific Areas (A) (2) (10153234), Scientific Research (B) (12557074, 13470184, 23300348, 15H04295), Scientific Research (C) (05807076, 09670931, 11670884, 16591204, 18591380, 20591493, 19 K08171) and Challenging Research (08877139, 26670556).

## AUTHOR CONTRIBUTIONS

Y.S. helped in performing the animal experiments. Y.S., H. T. and T.T. performed the rector operation for the neutron beam irradiation. K.T., M.S. and K.O. gave support in analyzing the data especially in terms of BNCT treatment. All authors read and approved the final manuscript.
